# Comparative Physiological, Biochemical, and Genetic Responses to Prolonged Waterlogging Stress in Okra and Maize Given Exogenous Ethylene Priming

**DOI:** 10.3389/fphys.2017.00632

**Published:** 2017-09-25

**Authors:** Emuejevoke Vwioko, Onyekachukwu Adinkwu, Mohamed A. El-Esawi

**Affiliations:** ^1^Department of Plant Biotechnology, Faculty of Life Sciences, University of Benin Benin, Nigeria; ^2^Botany Department, Faculty of Science, Tanta University Tanta, Egypt; ^3^The Sainsbury Laboratory, University of Cambridge Cambridge, United Kingdom

**Keywords:** waterlogging, ethylene priming, gene expression, *Abelmoschus esculentus*, *Zea mays*

## Abstract

Waterlogging is an environmental challenge affecting crops worldwide. Ethylene induces the expression of genes linked to important agronomic traits under waterlogged conditions. The ability of okra (*Abelmoschus esculentus* L. Moench.) and maize (*Zea mays* L.) given exogenous ethylene priming to tolerate prolonged waterlogged conditions was investigated in this study. The investigation was carried out as field experiments using 3 week-old plants grouped into four treatments; control, waterlogged plants, ethylene priming of plants before waterlogging, and ethylene priming of plants after waterlogging. Different growth parameters were recorded. Soil chemical and bacterial analyses were performed. The activity and gene expression of antioxidant enzymes were studied. The ethylene biosynthetic genes expression analysis and root anatomy of surviving okra plants were also carried out. Results revealed that okra and maize plants showed increase in their height under waterlogged conditions. Ethylene priming and waterlogged conditions induced early production of adventitious roots in okra and maize. Maize survival lasted between 5 and 9 weeks under waterlogging without reaching the flowering stage. However, okra survived up to 15 weeks under waterlogging producing flower buds and fruits in all treatments. Variable changes were also recorded for total soluble phenolics of soil. Cross sections of waterlogged okra roots showed the formation of a dark peripheral layer and numerous large aerenchyma cells which may have assisted in trapping oxygen required for survival. The activity and gene expression levels of antioxidant enzymes were studied and showed higher increases in the root and leaf tissues of okra and maize subjected to both waterlogging and ethylene priming, as compared to control or waterlogged condition. Quantitative RT-PCR analysis also showed that the ethylene biosynthetic gene expression levels in all okra and maize tissues were up-regulated and showed much higher levels under ethylene-treated waterlogged conditions than those expressed under control or waterlogged conditions at all time points. These results indicate that okra and maize tissues respond to the conditions of waterlogging and exogenous ethylene priming by inducing their ethylene biosynthetic genes expression in order to enhance ethylene production and tolerate the prolonged waterlogging stress. In conclusion, this study revealed that exogenously generated ethylene gas as a priming treatment before or after waterlogging could enhance waterlogging tolerance in maize and okra crops.

## Introduction

Waterlogging of upland communities in Southern Nigeria is a highly relevant environmental issue which is not well-addressed by agricultural experts. The flooding and waterlogging incidence of 2012 was economically devastating and many indigenous fishing and farming communities were sacked in Delta State (Nigeria). FAO (2002) also reported that over 10% of the global arable lands are affected by waterlogging and flooding annually. Hence, waterlogging has become a challenge to many agricultural zones of the world known for growing crops, including wheat, barley, maize, millet, sorghum, cassava, and leafy vegetables (Sayre et al., 1994). Waterlogged soil environments adversely affect the normal functioning of terrestrial plants and ecology (Setter and Waters, 2003). Widespread incidences of waterlogging are reported often in countries, including Pakistan, Nepal, India, Indonesia, Malaysia, Bangladesh and China, where the adverse effects are very pronounced in rice-wheat rotation farming systems (Samad et al., 2001).

Plants under waterlogged conditions are affected by gas exchange limitations, essential nutrient deficiencies and micronutrient toxicities such as Fe, Mn, and Cu (Setter et al., 2006). The shoot system of plants exhibit certain features, including wilting, premature yellowing of leaves (senescence), stunted plant height, epinasty, stem deformation, shoot length alteration, and leaf area reduction. Furthermore, crops including wheat show sterile floret and reduced grain yield and kernel weight (Hossain and Uddin, 2011). Root extension growth in terrestrial plants is arrested because the root tip is intolerant to hypoxic (low oxygen) or anoxic (absence of oxygen) conditions. Prolonged waterlogging will lead to alteration of chemical and physical properties of soil. These soil factors include pH, EC, hydraulic conductance, soil structure, porosity, and organics (Syversten et al., 1983; Setter et al., 2009). The oxygen-deficient soil environment leads to changes in composition and decomposition activities of microbes. The nutrient recycling process is hampered and nutrient deficiency conditions become eminent. The potential energy needed for nutrient uptake by plant roots comes primarily from aerobic respiration (Ferreira et al., 2008). In waterlogged soil, hypoxic, or anoxic conditions affect aerobic respiration and initiate anaerobiosis. In addition, synthesis and translocation of growth regulators, photosynthesis and carbohydrate partitioning are also negatively affected (Ferrer et al., 2005). These physiological impedances culminate in the reduced yield of crops under waterlogged conditions. In general, plant responses to waterlogged and flooding conditions are reported to include anatomical, physiological and molecular changes (Voesenek et al., 2006).

Ethylene is generated by plant tissues under abiotic stress such as drought and waterlogging (El-Esawi, 2016a,b,c). It is synthesized from methionine which forms *S*-adenosylmethionine. ACC synthase (ACS) then catalyzes the conversion of *S*-adenosylmethionine to 1-aminocyclopropane-1-carboxylate (ACC). ACC oxidase (ACO) then oxidizes ACC to generate ethylene (Geisler-Lee et al., 2010). Multigene families encode both ACS and ACO in several plant species such as maize (*Zea mays* L.) and okra (*Abelmoschus esculentus* L. Moench.). Among the well enunciated roles played by ethylene in waterlogged condition, induction of gene expression linked to leaf senescence, aerenchyma formation, adventitious roots, and epinasty are paramount (Jackson, 2008; Vidoz et al., 2010; Sasidharan and Voesenek, 2015) as morphological responses. These responses were observed with concomitant increase in endogenous ethylene synthesis in crops, including maize, barley, wheat, and soybean (He et al., 1994; Watkin et al., 1998; Drew et al., 2000). Exogenous ethylene treatment resulted in enhanced aerenchyma formation in rice (Takahashi et al., 2014). Under well-drained soil, aerenchyma formation is not observed in the root tissue of maize, whereas waterlogged condition induces aerenchyma formation in maize. Aerenchyma formation is attributed to the activity of ethylene in programmed cell death (PCD, Yamauchi et al., 2014).

Since the farming communities in Delta State (Nigeria) were substantially affected by the flood incidence of 2012, many farmlands were waterlogged for a period of at least 4 weeks and many crops generally did not tolerate this stress. In some of the riverine areas, farmlands were submerged for more than 2 weeks. Waterlogged conditions resulting from episodes of flooding in this region will reoccur as evidence of climate change. Therefore, the need to identify crops from the commonly grown crop species that can tolerate long periods of waterlogging has become foremost. Okra and maize are economically important food crops worldwide. The main objective of the current study was to assess okra and maize plants given exogenous ethylene priming for tolerating long periods of waterlogging. To achieve this objective, recording different plant growth parameters as well as root anatomy and soil chemical and bacterial analyses were conducted. Additionally, the activity and expression levels of antioxidant enzymes as well as the ethylene biosynthetic gene expression in okra and maize tissues were studied.

## Materials and methods

### Plant material

The seeds of okra variety Clemson spineless (produced by Technism and packed in France) and maize variety Oba-98, were used in this study.

### Preparation of soil samples and experimental pots

Composite soil sample was obtained from the Faculty of Agriculture Demonstration Farm, University of Benin, Nigeria. Five kilograms of soil was weighed into each experimental pot. Forty pots were prepared for the study. These pots were not perforated underneath so that water may be retained during the waterlogging experiment.

### Seed viability test, raising plants in nursery and transplanting

Test for seed viability was carried out following the floatation method. A large number of seeds were put in a bowl of water and allowed to stand for 10 min. Only seeds that sank down were taken as viable. The viable seeds were sown in a nursery to raise 2 week old plants that were later transplanted into prepared experimental pots for the waterlogged experiment. The transplanted crops were allowed to acclimatize to the pot environment for 1 week.

### Exposure of plants to ethylene gas

Based on preliminary experiments, ethylene priming of 350–400 ppm showed promising results. Okra and maize plants were, therefore, exposed to ethylene gas (375 ppm) for 1 h in the chamber. This was taken as ethylene priming of the young plants. For the purpose of ethylene priming, plants were divided into two groups; ethylene priming before waterlogging (EBW) or after waterlogging treatments, respectively.

### Experimental design and waterlogging conditions

The experimental plants were categorized into four groups; control (C), waterlogged (W), ethylene primed before waterlogging (EBW) and ethylene primed after waterlogging (EAW) for maize and okra used in this study. The plants grouped as control (C) were neither subjected to waterlogging condition nor ethylene priming. The group of waterlogged condition (W) consists of plants subjected to waterlogged condition without ethylene priming. The group of EBW was made up of plants given ethylene priming before subjecting to waterlogged condition. The group of EAW was made up of plants subjected to waterlogged condition, and after 2 days in this waterlogged condition, ethylene priming was carried out. The experiment pots were arranged as a completely randomized block design with five replicates. For waterlogging condition, each experimental pot was filled with water to cover the soil up to 1–2 cm above the soil surface, and this water level was maintained by adding water when necessary. Waterlogging condition was sustained for 4 weeks. The experimental data captured in the study were recorded in number of weeks counted from the first day plants were subjected to waterlogged condition.

### Growth traits measured

Growth traits measured included plant height, number of surviving leaves per plant, stem girth, number of adventitious roots formed, survival percentage, distance between first formed adventitious root and the soil level, extension of stem-root junction from the soil level, number of flower buds formed, and number of fruits produced.

### Soil chemical analysis

Analysis for soil factors, including pH, sulfate, phosphorus, organic carbon, nitrogen, manganese, iron and total soluble phenolics, were carried out following appropriate standard methods. The soil chemical analyses were done for the composite soil sample used for the study and for soil samples after plant growth. pH was determined in a soil-water slurry (ratio 1:3) (Ademoroti, 1996). Sulfate determination was carried out by a modification of the methods of Appiah and Ahenkorah (1989) and Ben Mussa et al. (2009). Phosphorus determination was performed using the method of Bray and Kurtz (1945). Organic carbon was conducted using Walkley-Black chromic acid wet oxidation method (Bremner and Jenkinson, 1960). Total nitrogen was performed using the Kjeldahl method (Bremner, 1960). Iron content was done using hydroxylamine and 1,10- phenanthroline procedure (Islam et al., 2009). Manganese was conducted using permanganate oxidation procedure (Islam et al., 2009). Total soluble phenolics analysis was performed using modification of citrate extraction procedure followed by Folin–Ciocalteau colorimetric method (Blum, 1997).

### Soil bacterial analysis

A weight of 10 g soil sample was measured into a beaker and mixed with 90 ml sterilized water. By serial dilution from the stock sample, 10^−4^ dilute solution was prepared. The pour plate technique was used for inoculation on a sterilized nutrient agar (NA), impregnated with antifungal agent, for the growth of bacterial isolates only. The plates were incubated at 37°C for 24–48 h. After incubation, total viable colonies were recorded for respective microbial isolates and expressed colony forming units per gram (cfu/g). The isolation, characterization and identification of bacterial isolates were performed following the reported methods (Buchanan and Gibbons, 1974).

### Root anatomy

The roots of harvested plants were cut and washed to prepare microscope slides to observe internal tissues. The root segments were embedded in paraffin wax and allowed to solidify. By clamping in the microtome, sections were cut and dewaxed. Eosin and hematoxylin stains were applied to the sections, respectively, to allow the cytoplasm and nucleus of cells to appear distinct under the microscope. Excess stains were washed off using increasing concentrations of ethanol sequentially at intervals before oven drying. After oven drying, the slides were ready for viewing and photographing.

### Antioxidant enzymes assay

Activities of Ascorbate peroxidase (APX) and catalase (CAT) enzymes were estimated in the leaf or root tissues of okra and maize following the protocol of Zhang and Kirkham (1996). Briefly, 0.25 g of leaf or root tissues were homogenized in 3 ml of a mixture (EDTA (0.2 mM), PBS (50 mM) and 1% PVP), and then centrifuged. The supernatant was used for measuring the absorbance at 240 nm (for CAT) or 290 nm (APX). Superoxide dismutase (SOD) activity was also estimated in the leaf or root tissues of okra and maize using the method of Bradford (1976). In brief, leaf or root plant tissues were homogenized with a phosphate buffer (0.2 M) then centrifuged. The supernatant was used for measuring the absorbance at 560 nm.

### RNA isolation, cDNA synthesis, and quantitative RT-PCR

Quantitative real-time PCR (qRT-PCR) analysis was carried out to assess the expression levels of antioxidant enzymes (APX, CAT, and SOD) in root or leaf tissues collected at several time points (3 days, 1 week, 2 weeks, 4 weeks) from the beginning of treatments (C, W, EBW, EAW). qRT-PCR analysis was also done to evaluate the expression levels of *ACS, ACO*, and ethylene receptor (*ETR2*) genes in the tissues of roots, hypocotyls and epicotyls of okra and maize collected at several time points from the beginning of treatments. Total RNA was isolated from these tissues using RNeasy Plant Mini kit (Qiagen), and RNase-Free DNase Set (Qiagen) was utilized to get rid of DNA. cDNA synthesis was performed using Reverse Transcription kit (Qiagen). Quantitative RT-PCR was conducted in triplicates (3 biological replicates and three technical repeats) using QuantiTect SYBR Green PCR kit (Qiagen) following the manufacturer's protocol. PCR amplification was accomplished under the following conditions: 95°C for 15 min; 50 cycles of 95°C for 30 s, 62°C for 30 s, 72°C for 2 min; and 72°C for 5 min. The gene specific-primer sequences used for amplification (Geisler-Lee et al., 2010; Hemavathi et al., 2011; Habib et al., 2016; Neta et al., 2016) are shown in Supplementary Table 1. Amplification specificity was then tested using melting curve analysis. UBIQUITIN (*UBQ1*, Chen et al., 2013) was used as a housekeeping gene, and the genes expression levels were measured using 2^−ΔΔCt^ method.

### Statistical analysis

Mean and standard deviation were calculated from the data collected. One-way analysis of variance was carried out. Significance of mean values was done using the Duncan multiple range (DMR) test.

## Results

### Plant height

**Figure 1:**
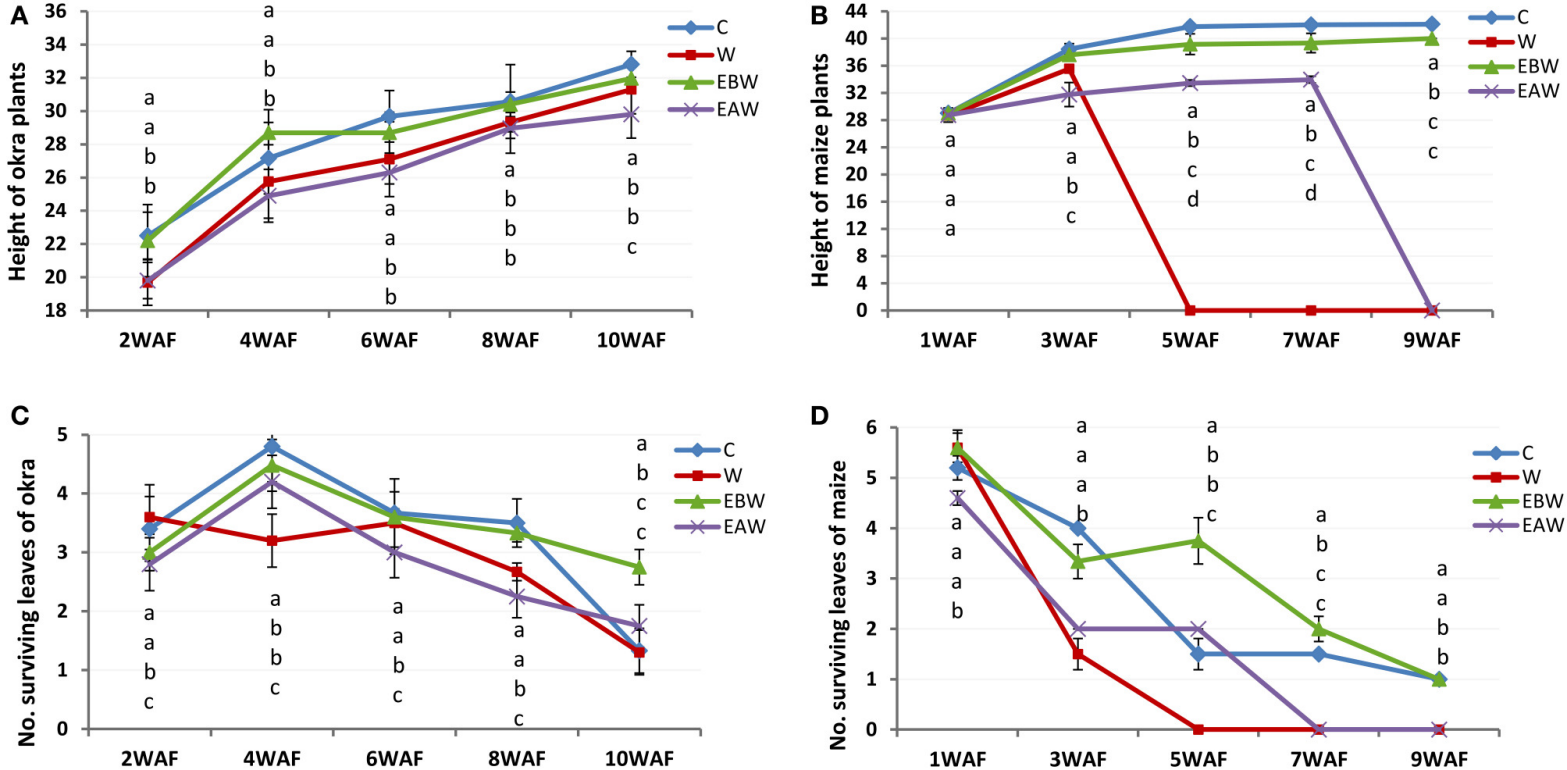
Height and number of surviving leaves of *Abelmoschus esculentus* and *Zea mays* plants subjected to waterlogged condition. **(A,C)** for Okra, and **(B,D)** for maize. Within the same WAF time point, values with similar alphabet do not differ significantly. Error bars represent SD, *n* = 5.

The values obtained for plant height indicated that the plants showed increase extension growth under waterlogged conditions. Okra plants showed high tolerance to waterlogging. Ten weeks under waterlogged conditions, the highest and lowest values for plant height were 32.81 and 29.80 cm for control and EAW treatments, respectively (Figure 1A; Supplementary Table 2). By 5 weeks under waterlogged conditions, all maize plants died under waterlogged (W) condition (Figure 1B; Supplementary Table 3). By 9 WAF, maize plants were observed only in EBW treatments.

### Number of surviving leaves

In okra plants, the number of leaves began to reduce immediately after 4WAF. Ten weeks under waterlogged condition, okra plants had a maximum of 3–4 healthy looking leaves per surviving plant (Figure 1C; Supplementary Table 4). The older leaves were lost first. The reduction of leaves in maize plants began 1 week after waterlogging. By 7WAF, all maize plants in two treatments (W, EAW) had lost all their leaves, and wilting of plants from the apical portions was apparent. Surviving maize plants in EBW treatments had average of two leaves (Figure 1D; Supplementary Table 5).

### Stem girth

**Figure 2:**
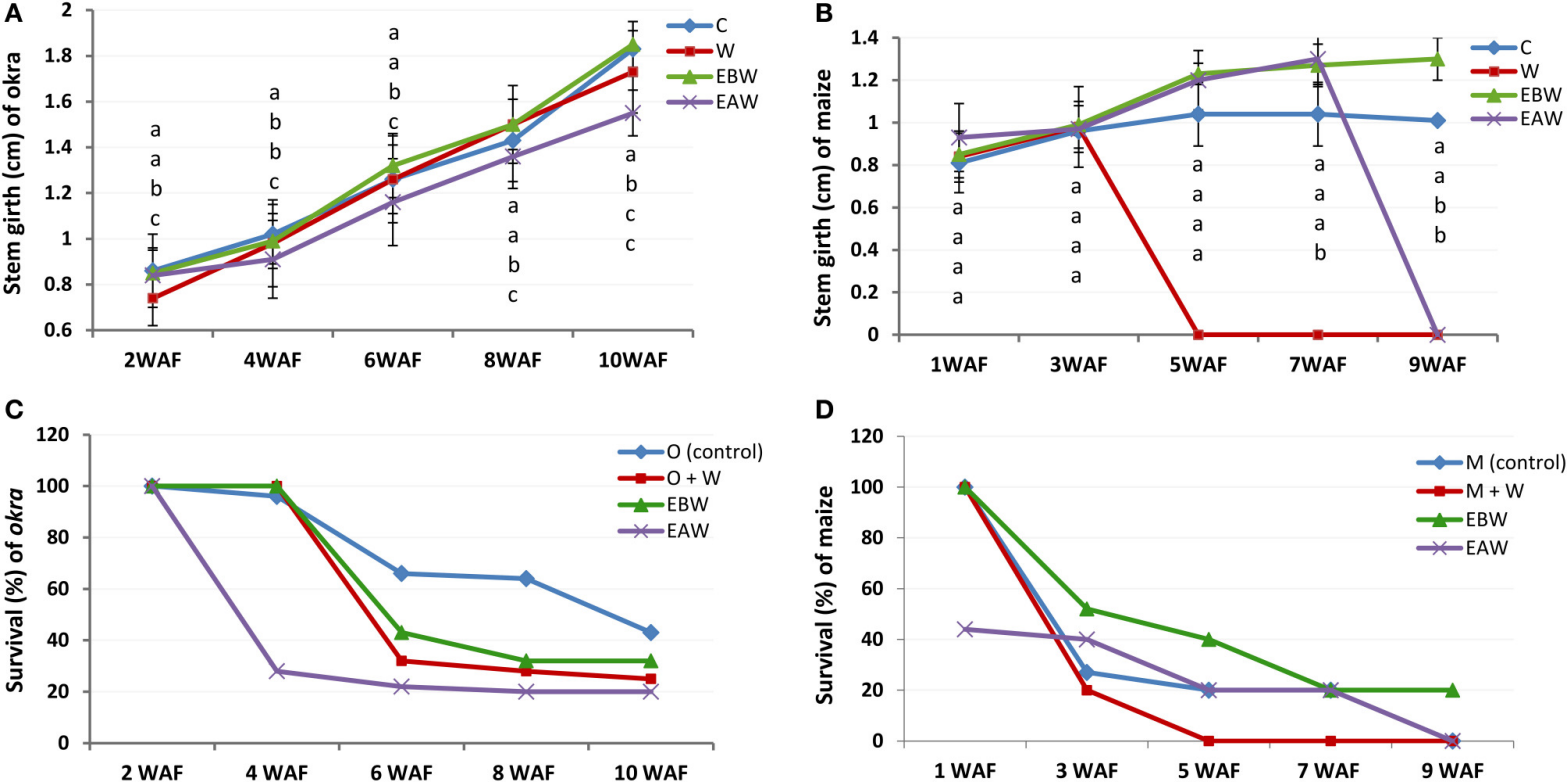
Stem girth (cm) and survival rate of *Abelmoschus esculentus* and *Zea mays* plants subjected to waterlogged condition. **(A,C)** for Okra, and **(B,D)** for maize. Within the same WAF time point, values with similar alphabet do not differ significantly. Error bars represent SD, *n* = 5.

Stem girth measurements for okra plants showed increase from 2WAF to 10WAF. The highest and lowest average values for stem girth of plants 10 weeks after flooding were 1.85 and 1.55 cm for EBW and EAW treated plants, respectively (Figure 2A; Supplementary Table 6). Maize plants (EBW) survived up to 9WAF and had an average value of 1.30 cm for stem girth (Figure 2B; Supplementary Table 7).

### Survival percentage

As the duration of waterlogged condition progressed, the number of surviving okra and maize plants reduced for the treatments. Ten weeks after flooding, the highest and lowest survival rates of okra plants were 43 and 20% for control and EAW, respectively (Figure 2C). Seven weeks after flooding, the average survival of maize plants was 20%. Only waterlogged maize plants survived under EBW treatment by 9WAF (Figure 2D).

### Number of adventitious roots

**Figure 3:**
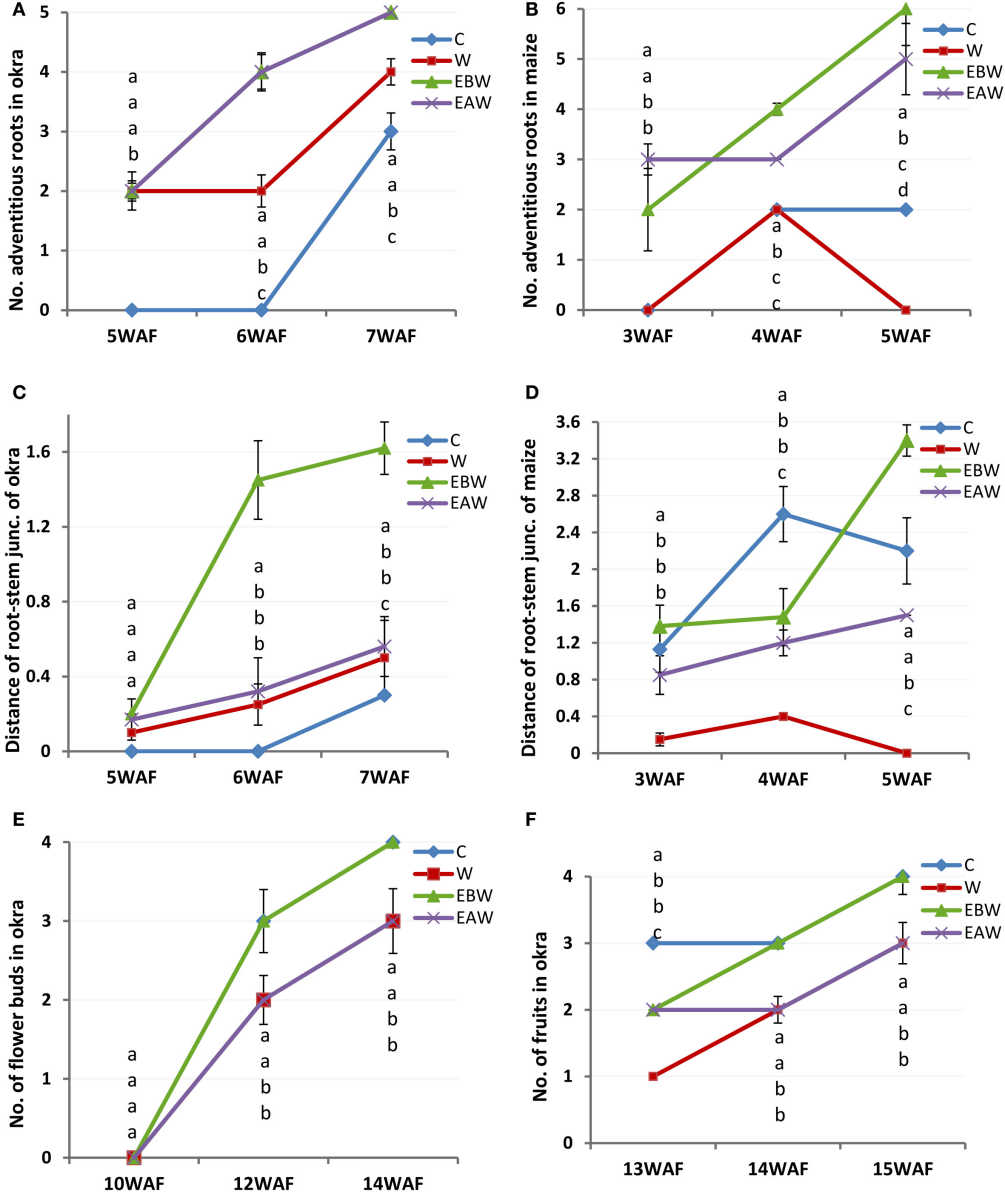
Number of adventitious roots, distance (cm) of root-stem junction and number of buds and fruits of *Abelmoschus esculentus* and *Zea mays* subjected to waterlogged condition. **(A,C,E,F)** for Okra, and **(B,D)** for maize. Within the same WAF time point, values with similar alphabet do not differ significantly. Error bars represent SD, *n* = 5.

Okra plants grown under EBW and EAW treatments exhibited the highest average number of adventitious root formed per plant (5 per plant). The lowest number, three per plant, was observed in the control plants. Adventitious roots were not recorded for control plants until 7WAF (Figure 3A; Supplementary Table 8). Maize plants under EBW and EAW treatments initiated adventitious roots earlier than others by 3WAF (Figure 3B; Supplementary Table 9). The average number of adventitious roots formed per maize plant grown under EBW and EAW treatments was higher than that of control plants until 5WAF.

### Extension of stem-root junction from the soil level

The extension of root-stem junction provides information on how the roots can extend above soil level to expose some portions of root to oxygen in order to ameliorate the anaerobic condition. Seven weeks after flooding of okra plants, the highest and lowest values for distance of root-stem junction above the soil level were recorded in EBW and control conditions, respectively (Figure 3C; Supplementary Table 10). Five weeks after flooding of maize plants, the lowest and highest values of root-stem junction above soil surface were 1.50 and 3.40 cm for EAW and EBW, respectively (Figure 3D; Supplementary Table 11). The maize plants under waterlogged (W) treatment died.

### Number of flower buds and fruits

All maize plants in this study failed to exhibit reproductive capacity or flower formation under the different treatments. Records of flower buds formed (Figure 3E; Supplementary Table 12) and fruits (Figure 3F; Supplementary Table 13) were taken for okra plants that tolerated waterlogged conditions. Okra plants under control and EBW treatments formed an average of four flower buds per plant by 14WAF. The average number of fruits per plant was 3 or 4 by 15WAF.

### Soil chemical and bacterial analyses

The changes in values of some selected soil parameters investigated were minimal (Table 1). The average phosphorus content of okra and maize plant grown under waterlogged conditions showed a reduction, when compared with the composite sample. Total soluble phenolics generally increased after plant growth. Values recorded for soil samples of C (maize), W (maize), EBW (maize), and C (okra) were >100 mg/Kg. Other soil samples gave lower values. Soil pH values generally ranged between 6 and 7. The bacterial isolates recorded for soil samples following maize and okra growth under waterlogged conditions, are also shown in Supplementary Table 14. The lowest and highest values for average bacterial counts were 6.4 × 10^4^ and 9.7 × 10^4^. *Micrococcus leutus* and *Micrococcus varians* were detected in 7 out of 8 soil samples. Other bacterial species include *Arthrobacter* sp, *Serratia* sp, *Pseudomonas* sp, and *Bacillus* spp.

**Table 1:** Values obtained for soil factors in composite soil sample before study and in the different experimental soil samples after plant growth under waterlogged condition.

**Soil parameters**	**Composite soil sample**	**C (maize)**	**W (maize)**	**EBW (Maize)**	**EAW (Maize)**	**C (okra)**	**W (Okra)**	**EBW (Okra)**	**EAW (Okra)**
pH (1:3)	6.49	6.32	6.14	6.77	6.44	6.67	6.47	6.88	6.74
Sulfate (mg/kg)	3.03 (0.21)	1.71 (0.03)	2.07 (0.02)	1.80 (0.04)	2.90 (0.15)	1.77 (0.17)	1.80 (0.17)	2.22 (0.08)	2.62 (0.09)
Phosphorus (mg/kg)	121.39 (0.56)	26.01 (0.18)	21.22 (0.16)	22.33 (0.05)	22.91 (0.07)	30.25 (0.10)	25.12 (0.09)	26.73 (3.35)	22.28 (0.06)
Organic carbon (%)	1.08 (0.88)	1.70 (0.03)	1.50 (0.02)	1.54 (0.05)	1.31 (0.03)	1.82 (0.04)	1.66 (0.03)	1.60 (0.02)	1.70 (0.07)
Total nitrogen (%)	0.88 (0.22)	0.55 (0.05)	0.55 (0.02)	0.59 (0.01)	0.55 (0.01)	0.38 (0.02)	0.28 (0.01)	0.57 (0.03)	0.35 (0.02)
Manganese (mg/kg)	4.10 (0.35)	2.36 (0.00)	3.48 (0.02)	4.01 (0.08)	3.49 (0.01)	2.30 (0.03)	1.98 (0.02)	2.89 (0.02)	2.76 (0.00)
Iron (mg/kg)	16.10 (0.20)	13.28 (0.03)	12.04 (0.02)	11.07 (0.02)	10.99 (0.02)	13.03 (0.03)	11.64 (0.00)	8.89 (0.02)	9.74 (0.01)
Total Soluble Phenolics (mg/kg)	49.00 (0.61)	121.70 (0.10)	163.20 (0.17)	159.80 (0.53)	93.20 (0.75)	167.23 (1.15)	22.24 (0.53)	70.70 (0.00)	57.13 (0.05)

*Values = mean (SD); C = control; W = under waterlogged; EBW = ethylene priming before waterlogged; EAW = ethylene priming after waterlogged*.

### Anatomy of okra roots

**Figure 4:**
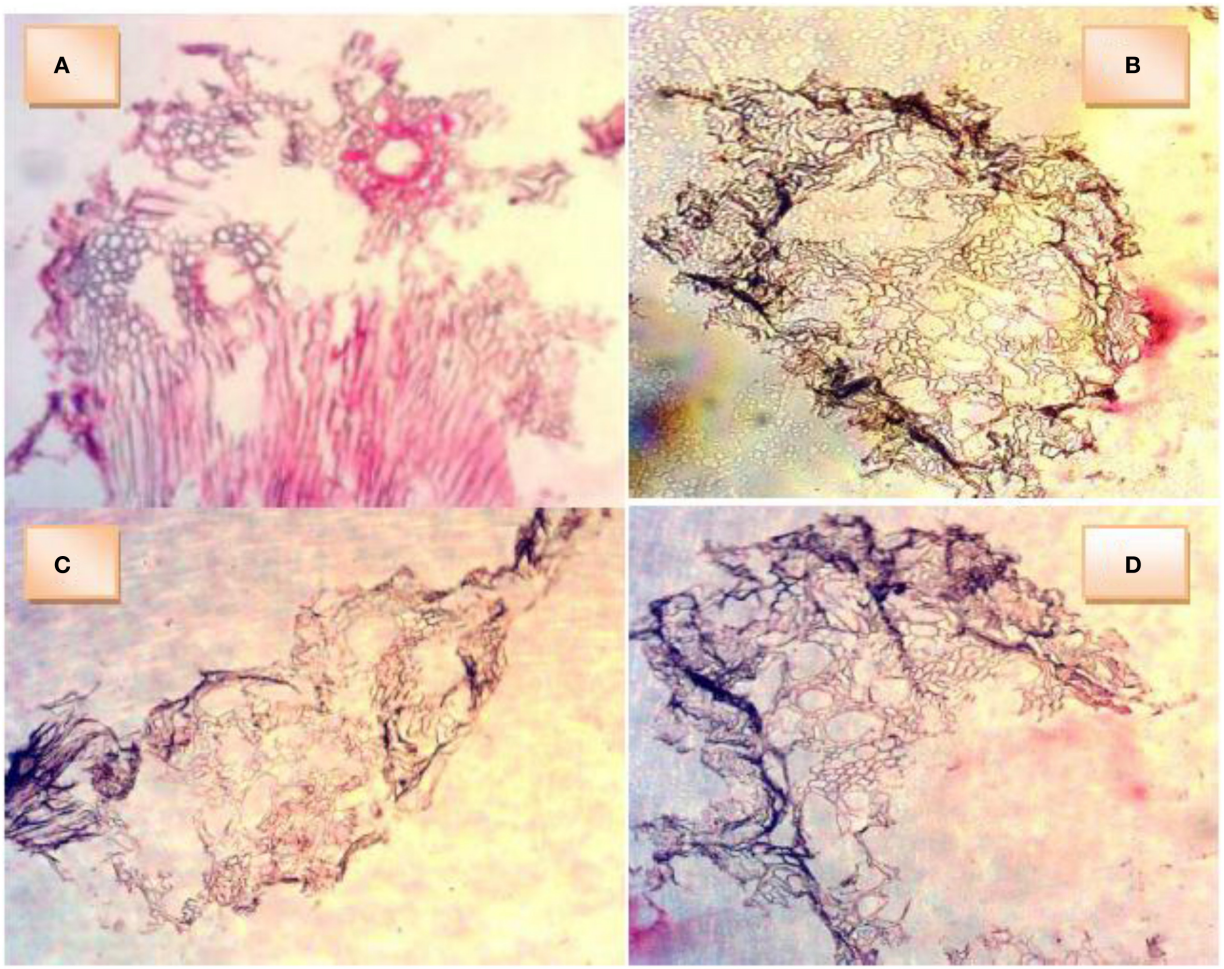
Cross sections of roots of *Abelmoschus esculentus* plants grown under waterlogged condition showing presence of aerenchyma cells and air cavities. **(A)** okra plant (control) without waterlogging, **(B)** okra plant given ethylene priming before waterlogging, **(C)** okra plant given ethylene priming after waterlogging, **(D)** okra plant under waterlogging only.

The cross section of okra roots showed a dark colored peripheral layer on the roots of waterlogged plants. This dark layer incompletely surrounded control root. All root sections showed the presence of air channels (lacunae) in the cortex. In Figure 4A (control), the cells around the air channels appear to possess thick walls. This control root was not submerged in water and as such the hypoxic condition would be milder than the others under water. Therefore, less change was exerted on the cellulose cell wall. Figure 4B (EBW) shows the air channels surrounded by cells whose walls indicate changes. The walls are thin but wall strengthening materials appear to be intact. More aerenchyma cells may be observe. Figure 4C (EAW) shows air channels surrounded by cells with wavy outlines of thin walls. This suggests an indication of collapsed wall strengthening materials. Figure 4D (W) shows air channels surrounded by cells with thin walls. Fewer aerenchyma cells may be seen.

### Activity and expression analyses of antioxidant enzymes

**Figure 5:**
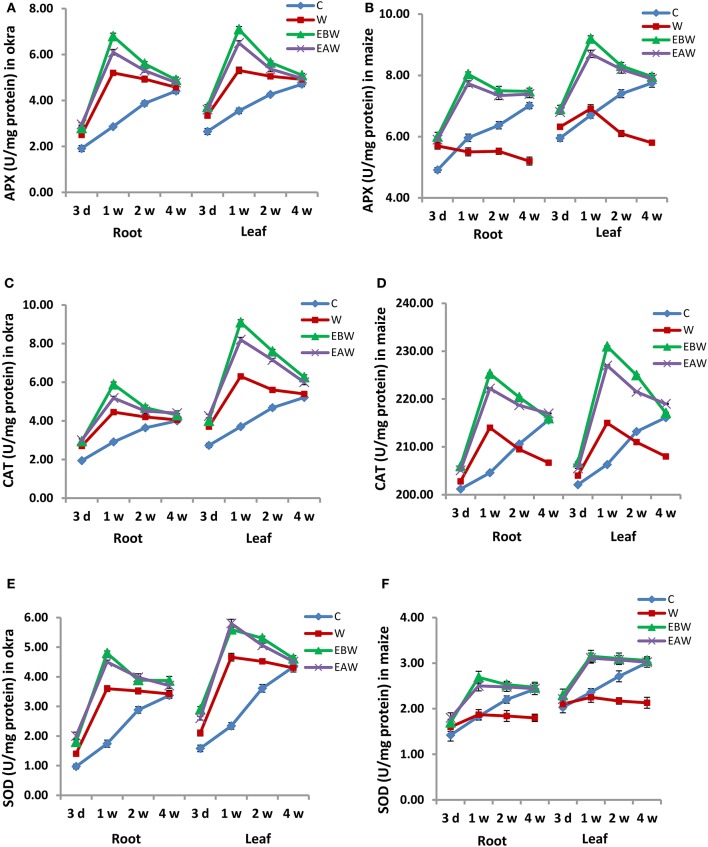
APX, CAT, and SOD enzyme activity in *Abelmoschus esculentus* and *Zea mays* subjected to waterlogged condition (d, day after flooding; w, week after flooding). **(A,C,E)** for Okra, and **(B,D,F)** for maize. Error bars represent SD, *n* = 4.

The effect of waterlogged conditions and exogenous ethylene priming on the activity and expression level of antioxidant enzymes (APX, CAT, and SOD) was studied in okra and maize root and leaf tissues collected at several time points (3 days, 1 week, 2 weeks, 4 weeks) from the onset of flooding. The activity of APX enzyme greatly increased in the root and leaf tissues of okra and maize subjected to waterlogging and ethylene priming (EBW, EAW), as compared to control (C) or waterlogged condition (W) (Figure 5). Additionally, the APX activity of okra exhibited a continuous increase under waterlogged condition (W) at all-time points, as compared to control (C).

Similarly, the activity of CAT enzyme greatly increased in the root and leaf tissues of okra and maize subjected to waterlogging and ethylene priming, as compared to control or waterlogged conditions (Figure 5). However, its activity in okra leaf was much higher than that in the root. On the other hand, the activity of SOD enzyme slightly increased in maize root and leaf tissues subjected to waterlogging and ethylene priming, whereas it highly increased in okra root and leaf under these conditions (Figure 5).

**Figure 6:**
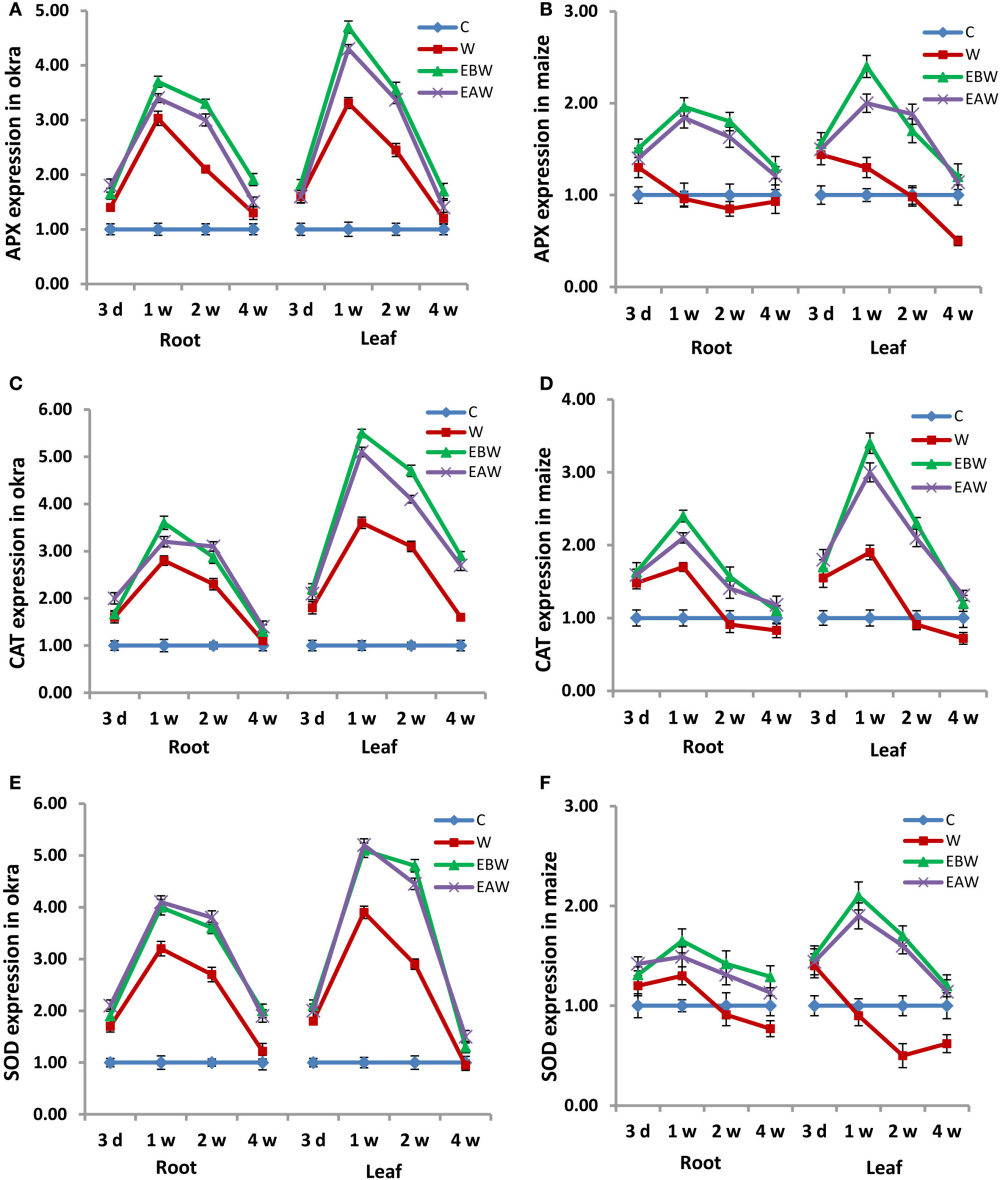
Expression levels of APX, CAT and SOD genes in *Abelmoschus esculentus* and *Zea mays* subjected to waterlogged condition (d, day after flooding; w, week after flooding). **(A,C,E)** for Okra, and **(B,D,F)** for maize. Error bars represent SD, *n* = 6.

Quantitative real-time PCR analysis was also conducted to evaluate the expression levels of these antioxidant enzymes in the root and leaf tissues of okra and maize subjected to waterlogging and ethylene priming, as well as to determine the correlation with the enzyme proteins. The expression analysis revealed that APX, CAT, SOD genes showed higher expression levels in the root and leaf tissues of okra and maize subjected to waterlogging and ethylene priming (EBW, EAW), as compared to control (C) or waterlogged condition (W) (Figure 6). mRNA expression results of these enzymes were in a complete concordance with their specific activities.

### Expression analysis of ethylene *ACS* and *ACO* genes

Quantitative real-time PCR analysis was carried out to study the effect of waterlogging conditions and exogenous ethylene treatment on the regulation of ethylene biosynthetic pathway genes in the roots, hypocotyls and epicotyls of okra and maize collected at several time points from the onset of flooding.

**Figure 7:**
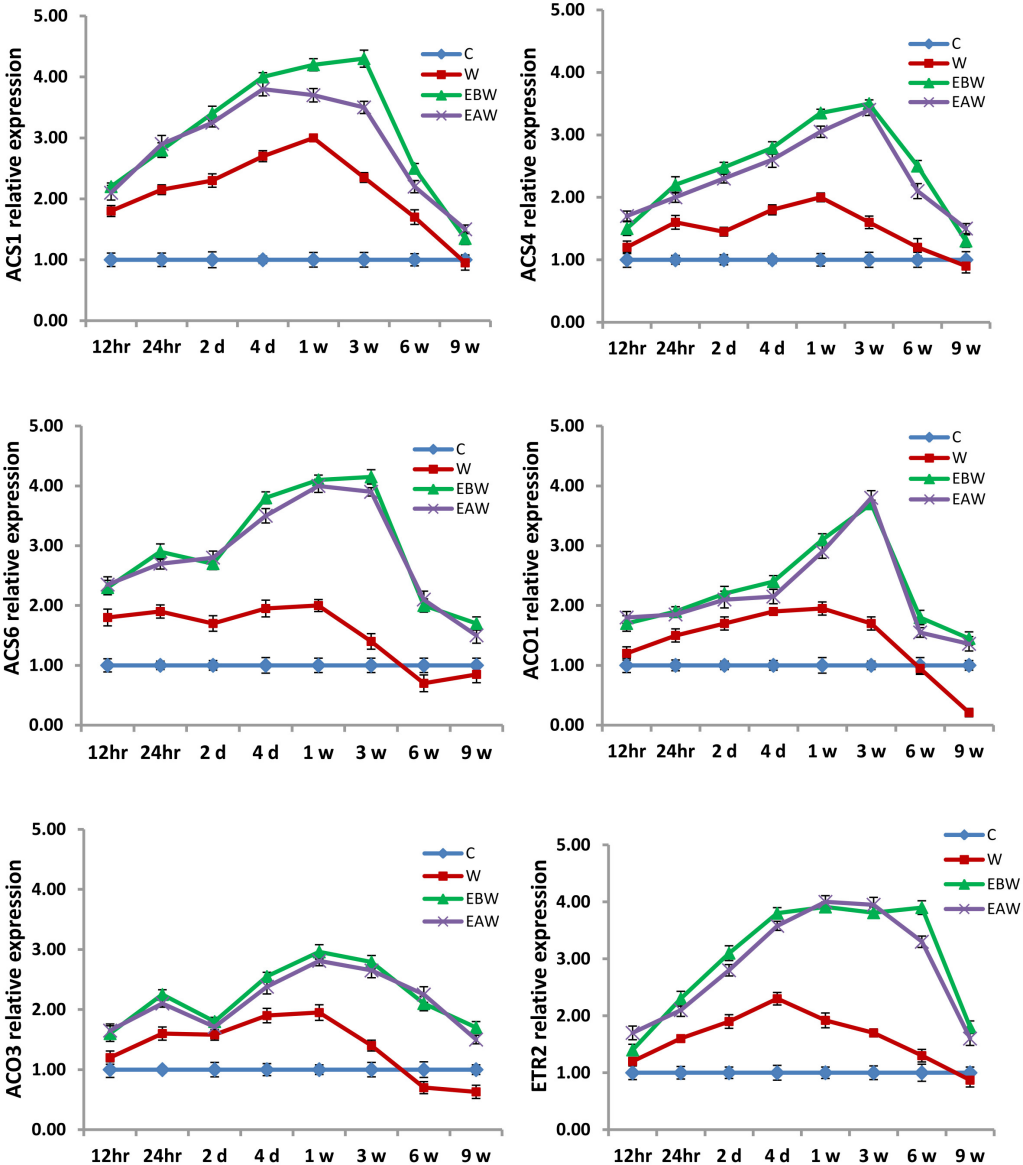
Expression levels of *ACS, ACO*, and *ETR2* genes analyzed in okra root subjected to waterlogged condition (d, day after flooding); w, week after flooding. Error bars represent SD, *n* = 6.

**Figure 8:**
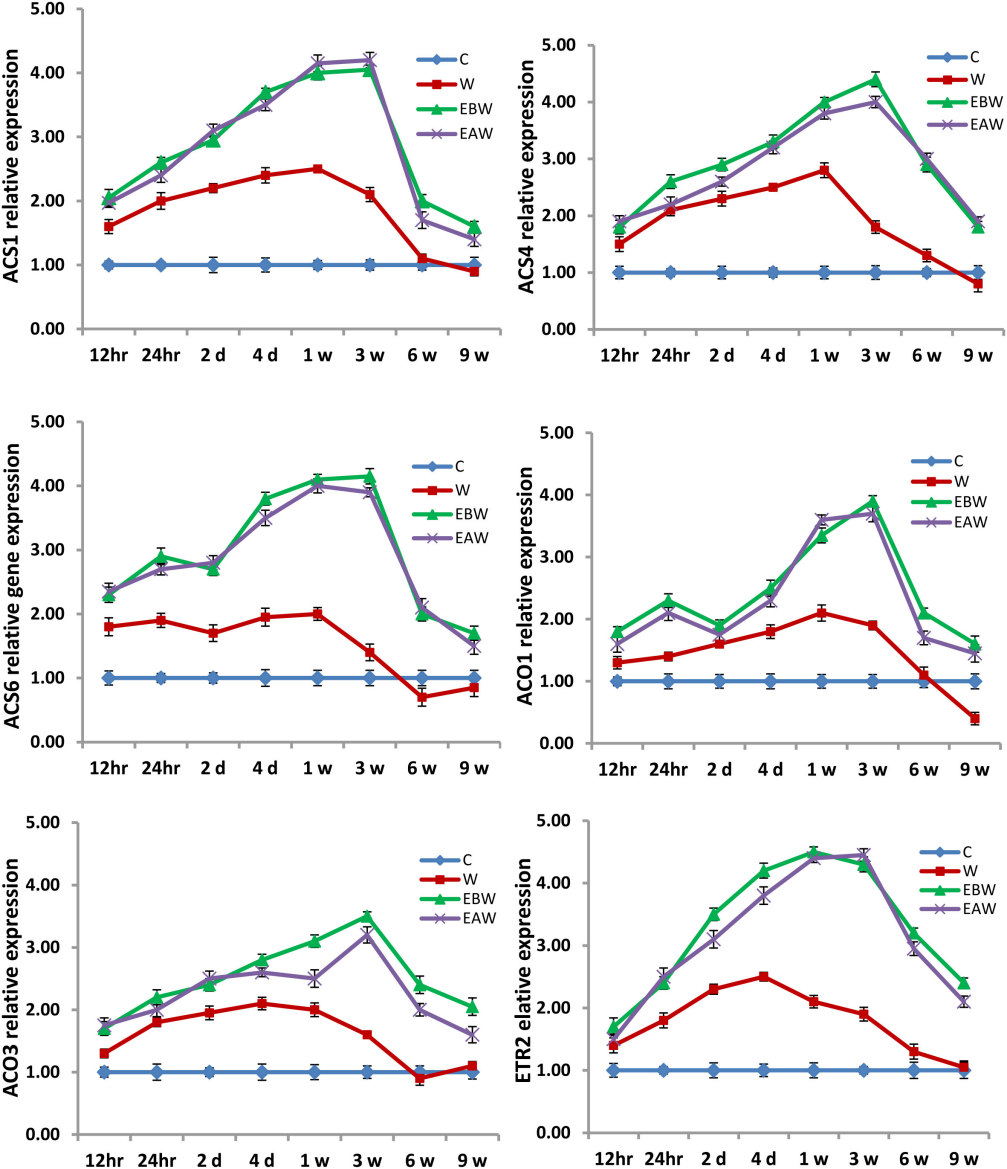
Expression levels of *ACS, ACO*, and *ETR2* genes analyzed in okra hypocotyl subjected to waterlogged condition (d, day after flooding; w, week after flooding). Error bars represent SD, *n* = 6.

**Figure 9:**
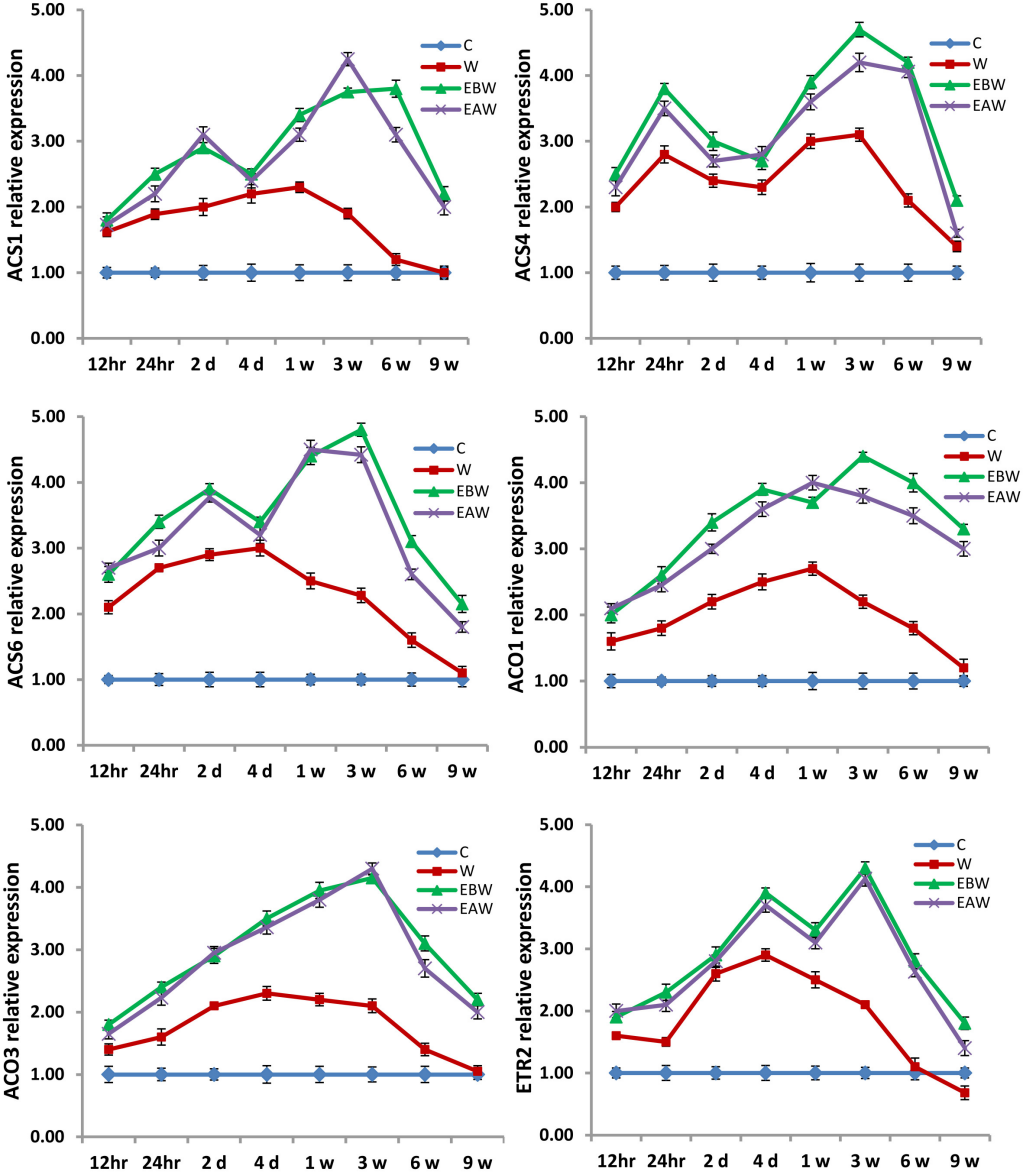
Expression levels of *ACS, ACO*, and *ETR2* genes analyzed in okra epicotyl subjected to waterlogged condition (d, day after flooding; w, week after flooding). Error bars represent SD, *n* = 6.

The expression levels of *ACS1, ACS4, ACS6, ACO1, ACO3*, and *ETR2* genes were assessed in okra roots, hypocotyls and epicotyls collected at several time points (12 h, 24 h, 2 days, 4 days, 1 week, 3 weeks, 6 weeks, 9 weeks) from the onset of flooding (Figures 7–9). Interestingly, waterlogging and exogenous ethylene priming (EBW, EAW) significantly induced the expression levels of all *ACS, ACO*, and *ETR2* genes in all okra tissues until 9 weeks of waterlogging, as compared to the control (C) or waterlogging (W) conditions (Figures 7–9).

**Figure 10:**
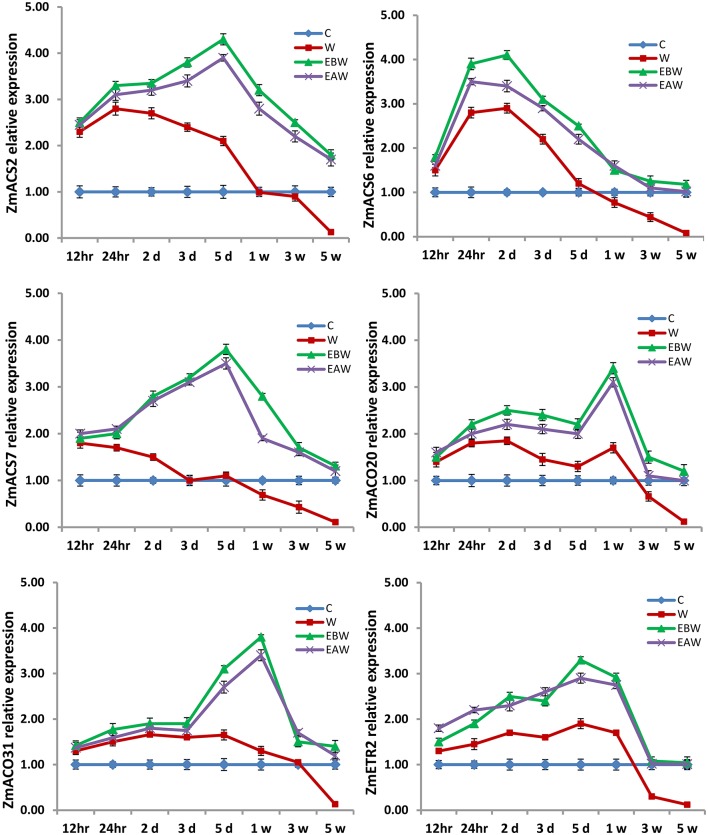
Expression levels of *ZmACS, ZmACO*, and *ZmETR2* genes analyzed in maize root subjected to waterlogged condition (d, day after flooding; w, week after flooding). Error bars represent SD, *n* = 6.

**Figure 11:**
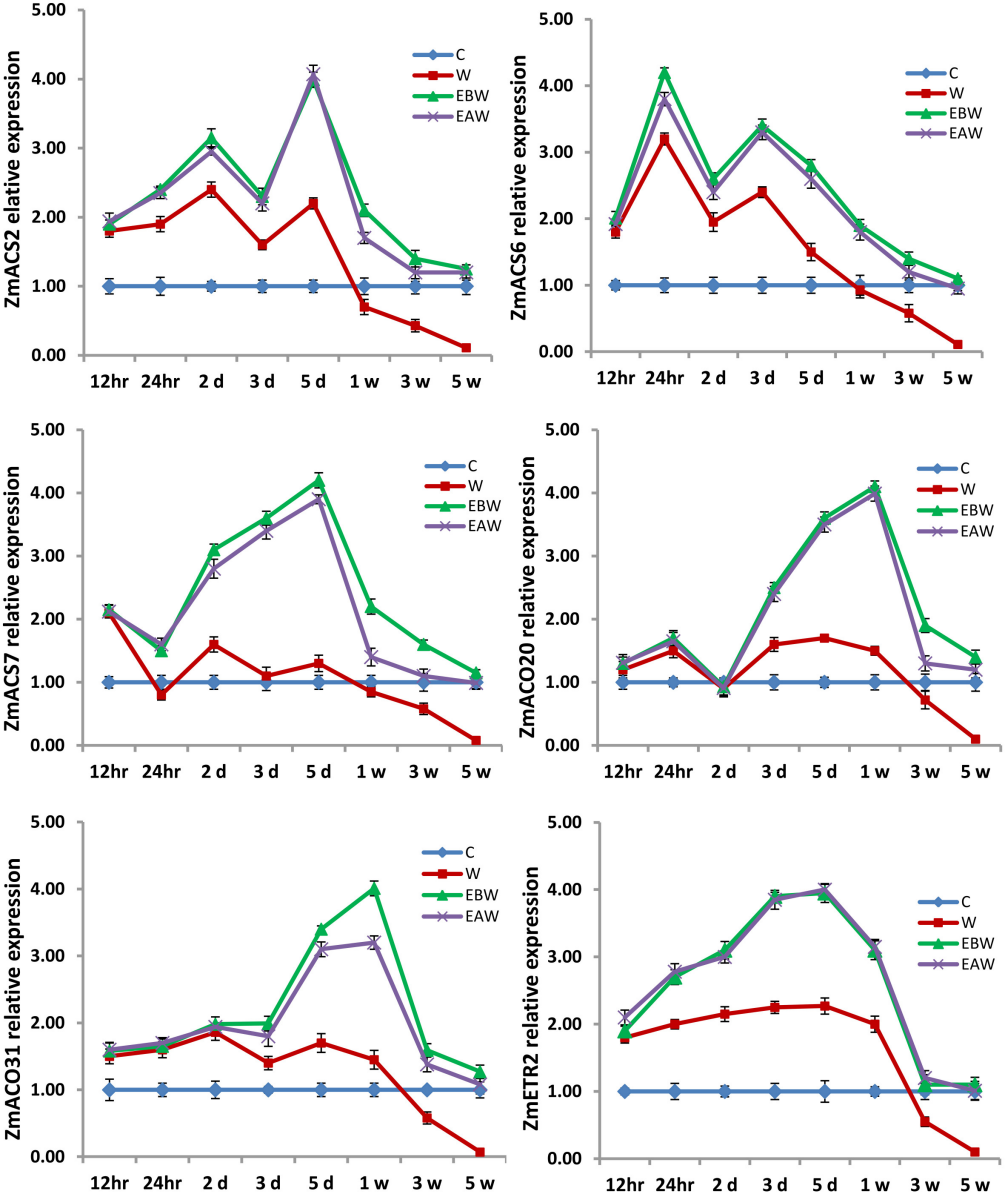
Expression levels of *ZmACS, ZmACO*, and *ZmETR2* genes analyzed in maize hypocotyl subjected to waterlogged condition (d, day after flooding; w, week after flooding). Error bars represent SD, *n* = 6.

**Figure 12:**
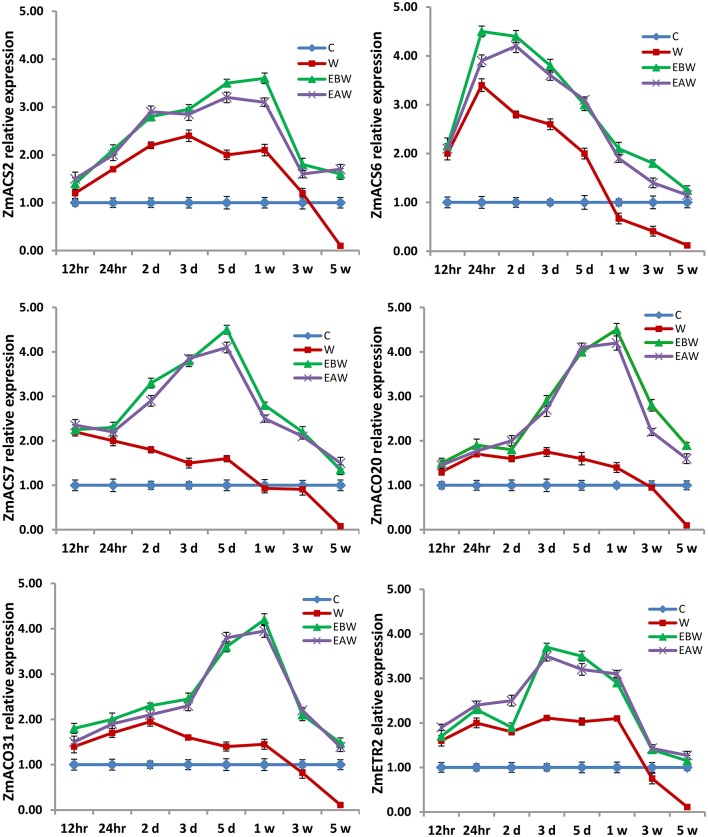
Expression levels of *ZmACS, ZmACO*, and *ZmETR2* genes analyzed in maize epicotyl subjected to waterlogged condition (d, day after flooding; w, week after flooding). Error bars represent SD, *n* = 6.

The expression levels of *ZmACS2, ZmACS6, ZmACS7, ZmACO20, ZmACO31*, and *ZmETR2* genes were also studied in maize roots, hypocotyls and epicotyls collected at several time points (12 h, 24 h, 2 days, 3 days, 5 days, 1 week, 3 weeks, 5 weeks) from the onset of flooding (Figures 10–12). Interestingly, waterlogging and exogenous ethylene priming (EBW, EAW) induced the expression levels of *ACS, ACO*, and *ETR2* genes in all maize tissues until 3 or 5 weeks of waterlogging, as compared to the control (C) or waterlogged conditions (W) (Figures 10–12). The results indicate that the high expression levels of *ACS, ACO*, and *ETR2* genes lasted for longer periods after waterlogging and ethylene priming in okra, as compared to maize.

## Discussion

Water is indispensable to the survival of plants as it supports plant growth and functions. However, flooding or waterlogging threatens plants. The negative impact of flooding leaves the society and environment devastated. Plant biodiversity, distribution of natural species and worldwide food production decline because terrestrial plant species, including cultivated crops, are sensitive to flood conditions (Normile, 2008). The harmful effect of flooding is attributed to the fact that gas diffusion in water, as a medium of transport within living systems, is too low to allow terrestrial plants survival for a long time. Two vital plant processes, respiration and photosynthesis, are affected (Sasidharan and Voesenek, 2015). Sasidharan and Voesenek (2015) reported that the energy crisis resulting from hampered aerobic metabolism leads to an imbalance between consumption and production, resulting in plant mortality. Waterlogging describes only the situation where the soil and plant roots are covered by water. In this study, the values for plant height as growth parameter evaluated were higher in control plants than in plants subjected to waterlogged condition. The waterlogged conditions stimulated early formation of adventitious roots of okra plants in this study. Generally, the plants suffered loss of leaves in the process of surviving the condition.

Waterlogged conditions reduce available oxygen to plant root cells simply by displacement of air pockets in the soil. Therefore, there is a steep oxygen gradient between root and shoot portions. Roots in waterlogged soils suffer rapid oxygen depletion due to respiration both of the roots and the root-associated microbiome (Vashisht et al., 2011). These waterlogged roots switch to the inefficient anaerobic fermentation, consuming available carbohydrate reserves for the generation of needed ATP to remain alive and functioning. As the hypoxic or anoxic situation continues, impaired membrane integrity, starvation and diffusion of phytotoxic compounds into the root cells combine to hinder root growth and function (Sauter, 2013). Since the roots cannot transport water and nutrients efficiently under hypoxic or anoxic conditions, shoot functions are affected and visible symptoms such as wilting, senescence, and death may be observed (Sasidharan and Voesenek, 2015).

In sensitive genotypes, waterlogged conditions may cause induction and initiation of crop tolerance features or adaptive traits that may improve aeration and ameliorate root hypoxia or anoxia in order to preserve root function and plant survival. Those adaptive features observed included development of a lignin/suberin barrier in the root to reduce oxygen loss and commit its transport to the tip of the root (Shiono et al., 2011), enhanced development of aerenchyma cells to promote root aeration (Takahashi et al., 2014) and development of adventitious roots rich in aerenchyma (Sauter, 2013). Maize and okra plants are sensitive to waterlogged conditions. Both species developed adventitious roots. The root extension growth also enabled the root-stem junction to rise above the soil surface, exposing portions of the root to molecular oxygen aerially. This should be considered as another feature to survive the waterlogged conditions.

Phytohormones are important to plants in signals and responses to stress. Many plant responses are based on the sensitivity of the tissues involved. Many cells of higher plants can carry out synthesis of ethylene in low amount (Abeles, 1992). Root hypoxia (low oxygen) or anoxia (oxygen deficient) induced by waterlogging limits the conversion of ACC to ethylene because the ACC oxidase needs molecular oxygen to complete the synthesis of ethylene (Jackson, 2008). Hypoxia or anoxia favors the accumulation of ACC in flooded roots, which is transported to the shoot, where it is usually converted to ethylene. Bradford and Yang (1980) classified ACC as the primary signal transferred from roots to shoots during the early period of hypoxia. The conversion of ACC to ethylene in the presence of molecular oxygen triggers the development of adaptive systemic responses by shoots of waterlogged plants and these include aerenchyma cells and adventitious root formation (Fukao and Bailey-Serres, 2008; Rajhi et al., 2011; Yamauchi et al., 2014). Molecular oxygen, generated from photosynthetic activities of the aerial shoot, diffuses downward to the aerenchyma cells and aerenchyma-rich adventitious roots under hypoxic or anoxic condition, as an amelioration mechanism in mesophytes (Irfan et al., 2010). Rajhi et al. (2011) stated that application of ethylene induced the development of aerenchyma in maize plants. Under hypoxia, Takahashi et al. (2014) observed further augmentation of aerenchyma development in rice, which is dependent on treatment with ethylene or ACC. Degree of aerenchyma induction and formation by endogenous ethylene treatment varies between species. Aerenchyma formation is completed as a genetically programmed death process in the cortex of roots, which high levels of reactive oxygen species (ROS) are capable of triggering (Steffens et al., 2011; Yamauchi et al., 2014). ROS is one component of the ethylene-mediated signaling network (Sasidharan and Voesenek, 2015). Separately, ROS or ethylene induces ectopic cell death only, whereas ethylene-mediated ROS is important in the formation of aerenchyma. Aerenchyma, formed in the root cortex, provides aeration, and enhances survival. The plants under waterlogged conditions developed more adventitious roots than the control. Also, the plants given ethylene priming produced higher number of adventitious roots per plant. This supports the hypothesis that plants develop morphological adaptative features under waterlogged conditions.

The waterlogging-induced production of crop adventitious roots, which functionally replace the damaged soil-borne roots, improves the shoot-root diffusion of gases (Sasidharan and Voesenek, 2015). Ethylene has been reported to be important in adventitious root formation. McNamara and Mitchell (1989) concluded that auxin interacts with ethylene to induce the development of adventitious roots, but the role of ethylene may differ relying on the plant species (Vidoz et al., 2010). Ethylene induces an increase in auxin-sensitivity of root forming tissue during adventitious root formation in waterlogged *Rumex palustris* (Visser et al., 1996), whereas the induction of adventitious root production during waterlogging requires ethylene perception by the Never Ripe receptor in tomato (Vidoz et al., 2010). The transport of ACC from hypoxia or anoxia roots to the shoot stimulates ACC synthase genes to make ACC synthase enzyme available to complete the biosynthesis of ethylene in the molecular oxygen-mediated process. The elevated level of ethylene in the stem reprogrammes auxin transport in the shoot, directing the flow of auxin toward the submerged stem to initiate the growth of adventitious roots. Adventitious root production is hindered if auxin transport is inhibited (Vidoz et al., 2010).

The present study has shown that okra survived better than maize under waterlogged condition. The okra plants were able to flower and fruit, whereas maize plants did not survive beyond 9WAF and were unable to flower and form fruits during this period. Some maize plants given EBW were the ones that survived up to 9WAF. The highest population of soil bacterial flora in a soil sample after plant growth was detected in EBW (maize), along with an absence of *Micrococcus* spp. The survival of maize plants under EBW treatment indicates that EBW was beneficial to plants. The changes in soil factors following plant growth of maize and okra did not reflect a major difference between the two crops. The results of cross-sections of okra roots showed presence of aerenchyma cells. The root tissues of okra plants possess large air channels which may have assisted in trapping oxygen useful for survival. Formation of aerenchyma improves the porosity of roots (Videmsek et al., 2006). Differences in porosity of roots of a genotype have been reported (Thomson et al., 1990) and this can be linked to the size and number of aerenchyma present. Porosity allows more gas to be present within the internal tissues of root. Justin and Armstrong (1987) reported that since porosity promotes internal movement of gases, plant roots adapted to anaerobic conditions should possess higher porosity as a characteristic. The formation of the dark peripheral layer (suspected to be impervious) and numerous large aerenchyma by waterlogged roots of okra suggest promising support for tolerance of anaerobic conditions. In addition, exogenous ethylene priming provides a beneficial influence on growth of okra under waterlogged conditions.

In the current study, the specific activities and transcript levels of three antioxidant enzymes were also studied in okra and maize root and leaf tissues subjected to waterlogging and ethylene priming. APX, CAT, and SOD enzyme activities increased in the root and leaf tissues of okra and maize subjected to both waterlogging and ethylene priming, as compared to control or waterlogged condition, indicating the efficient role of antioxidant enzymes in tolerating waterlogging stress. mRNA expression levels of these enzymes revealed a positive correlation with their specific activities.

In the present study, qRT-PCR analysis also showed that the expression levels of *ACS, ACO*, and *ETR2* genes in all okra and maize tissues were up-regulated and showed much higher levels under EBW and EAW treatments than those expressed under control or waterlogged conditions at all-time points. This indicates that okra and maize tissues respond to conditions of waterlogging and exogenous ethylene priming by inducing their ethylene biosynthetic genes expression in order to enhance ethylene production and tolerate the prolonged waterlogging stress. In conclusion, this study revealed that exogenously generated ethylene gas as a priming treatment before or after waterlogging could enhance waterlogging tolerance in maize and okra crops.

## Author contributions

ME and EV designed the study. ME, EV, and OA carried out the experiments, analyzed the data, and wrote the manuscript. All authors revised and approved the final manuscript.

### Conflict of interest statement

The authors declare that the research was conducted in the absence of any commercial or financial relationships that could be construed as a potential conflict of interest.
