# Supplementary material for: Comparative Physiological, Biochemical, and Genetic Responses to Prolonged Waterlogging Stress in Okra and Maize Given Exogenous Ethylene Priming
Source: Front Physiol. 2017 Sep 25;8:632. doi: 10.3389/fphys.2017.00632 (PMC5622204; doi:10.3389/fphys.2017.00632)
Supplement: Supplementary file 1 [file Table1.PDF]

Supplementary Tables

Comparative Physiological, Biochemical and Genetic Responses to Prolonged Waterlogging Stress in Okra and Maize Given Exogenous Ethylene Priming

Emuejevoke Vwioko[1], Onyekachukwu Adinkwu[1] and Mohamed A. El-Esawi[2,3*]

[1]Department of Plant Biotechnology, Faculty of Life Sciences, University of Benin, Benin, Nigeria.

[2]Botany Department, Faculty of Science, Tanta University, 31527 Tanta, Egypt.

[3] The Sainsbury Laboratory, University of Cambridge, Cambridge, United Kingdom.

*Corresponding author, E-mail: mohamed.elesawi@science.tanta.edu.eg

Supplementary Table 1. Primer sequences of genes analyzed using qRT-PCR analysis.

Gene	NCBI accession number	Primer sequence (5′–3′)
1. Maize:		
CAT (cat1)	NM_001254879	F: CTGAACGTGTTGTGCATGCA<br>R: TAGATCCTTCGTCGCATGGC
SOD (sod4)	NM_001112234	F: TGGAGCACCAGAAGATGA<br>R: CTCGTGTCC ACCCTTTCC
APX (apx2)	NM_001112030	F: TGAGCGACCAGGACATTG<br>R: GAGGGCTTTGTCA CTTGGT
ZmACS2	AY359585	F: ATCGCGTACAGCCTCTCCAAGGA<br>R: GATAGTCTTTTGTCAACCATCCCATAGA
ZmACS6	AY359587	F: AGCTGTGGAAGAAGGTGGTCTTCGAGGT<br>R: AGTACGTGACCGTGGTTTCTATGA
ZmACS7	NM_001152929	F: ATCGCGTACAGCCTCTCCAAGGA<br>R: CAACGTCTCTGTCACTCTGTGTAATGT
ZmACO20	NM_001111765	F: CTCATCCTGCTGCTCCAGGACGAC<br>R: TCCACGATACACGCATAACCACCGT
ZmACO31	NM_001111764	F: CTCGTCTTCGATCAATTCCCAAGT<br>R: ATAGCAAAGAGGGCAACTAGCTAGT
ZmETR2	NM_001111382	F: GCCCACGTGTAGACGGTTAT<br>R: AACCAGGCTTCATTCAGACG
UBQ1	AT3G52590	F: TTCCTTGATGATGCTTGCTC<br>R: TTGACAGCTCTTGGGTGAAG
**2. Okra:**		
CAT	AY442179	F: TGCCCTTCTATTGTGGTTCC<br>R: GATGAGCACACTTTGGAGGA
APX	AB041343	F: ACCAATTGGCTGGTGTTGTT<br>R: TCACAAACACGTCCCTCAAA
SOD	AF354748	F: GTTTGTGGCACCATCCTCTT<br>R: GTGGTCCTGTTGACATGCAG
ACS1	DQ174259	F: CGTCGATCGGTCCTGTTTAT<br>R: TTTGCCACACGTATTTTCCA
ACS4	JF508505	F: CAGAGTGAAGGGCTTGCTCA<br>R: TCCATCTCGGCTGCAAATGT
ACS6	DQ122174	F: GAGGTGGAGAACAGGGGTTG<br>R: CTGCTCCTTGCTCTCCACAA
ACO1	DQ116442	F: GCAGTGGAGTTGGAGAAGCT<br>R: CTCCACGGTCTTCATGGCTT
ACO3	DQ116444	F: ATGGAGCAAATCAAGGATGC<br>R: GCCTTGCTTGCTACCAGTTC
ETR2	XM_016859813	F: GCTTCAGATGCCCGAGTTAG<br>R: GACTGGCTTTCGAACGACTC

Supplementary Table 2. Height of *Abelmoschus esculentus* plants subjected to waterlogged condition

Treatment	2WAF	4WAF	6WAF	8WAF	10WAF
Control	22.50 (1.41)^{b}	27.16 (2.15)^{b}	29.68 (1.55)^{b}	30.57 (2.22)^{b}	32.81 (0.78)^{c}
W	19.68 (1.37)^{a}	25.76 (2.21)^{a}	27.10 (2.25)^{a}	29.33 (0.58)^{a}	31.30 (1.47)^{b}
EBW	22.20 (2.17)^{b}	28.70 (1.38)^{b}	28.70 (1.23)^{b}	30.40 (0.74)^{a}	31.97 (0.83)^{b}
EAW	19.80 (1.09)^{a}	24.90 (1.59)^{a}	26.30 (0.69)^{a}	28.96 (1.50)^{a}	29.80 (1.43)^{a}

Values = mean (SD). C = control; W= waterlogged; EBW=ethylene before waterlogged; EAW=ethylene after waterlogged. WAF= Weeks after flooding. Plants were three weeks old when treatment was applied and flooding introduced and sustained for four weeks. The values with similar alphabet as superscript on the same column do not differ significantly.

Supplementary Table 3. Height (cm) of *Zea mays* plants subjected to waterlogged condition

Treatment	1WAF	3WAF	5WAF	7WAF	9WAF
Control	29.02 (0.61)^{a}	38.43 (0.81)^{c}	41.75 (0.35)^{d}	42.00 (0.34)^{d}	42.11 (0.29)^{d}
W	28.66 (0.41)^{a}	35.55 (0.35)^{b}	0.00 (0.00)^{a}	0.00 (0.00)^{a}	0.00 (0.00)^{a}
EBW	29.02 (0.61)^{a}	37.60 (0.87)^{c}	39.16 (1.52)^{c}	39.33 (1.42)^{c}	40.00 (0.00)^{b}
EAW	28.74 (1.04)^{a}	31.75 (1.77)^{a}	33.45 (0.45)^{b}	33.95 (0.50)^{b}	0.00 (0.00)^{a}

Values = mean (SD). C= control; W=waterlogged; EBW=ethylene before waterlogged; EAW=ethylene after waterlogged. WAF= Weeks after flooding. Plants were three weeks old when treatment was applied and flooding introduced and sustained for four weeks. The values with similar alphabet as superscript on the same column do not differ significantly. The value, 0.00 indicates no plant available.

Supplementary Table 4. Number of surviving leaves present in *Abelmoschus esculentus* plants subjected to waterlogged condition

Treatment	2WAF	4WAF	6WAF	8WAF	10WAF
Control	3.40 (0.55)^{c}	4.80 (0.60)^{c}	3.67 (0.58)^{c}	3.50 (0.41)^{c}	1.33 (0.38)^{a}
W	3.60 (0.55)^{c}	3.20 (0.45)^{a}	3.50 (0.53)^{b}	2.67 (0.15)^{b}	1.30 (0.38)^{a}
EBW	3.00 (0.31)^{b}	4.48 (0.44)^{b}	3.60 (0.14)^{c}	3.33 (0.15)^{c}	2.75 (0.30)^{c}
EAW	2.80 (0.45)^{a}	4.20 (0.45)^{b}	3.00 (0.43)^{a}	2.25 (0.36)^{a}	1.75 (0.36)^{b}

Values = mean (SD). C = control; W= waterlogged; EBW=ethylene before waterlogged; EAW=ethylene after waterlogged. WAF= Weeks after flooding. Plants were three weeks old when treatment was applied and flooding introduced and sustained for four weeks. The values with similar alphabet as superscript on the same column do not differ significantly.

Supplementary Table 5. Number of surviving leaves present in *Zea mays* plants subjected to waterlogged condition

Treatment	1WAF	3WAF	5WAF	7WAF	9WAF
Control	5.20 (0.24)^{b}	4.00 (0.00)^{b}	1.50 (0.31)^{b}	1.50 (0.00)^{c}	1.00 (0.00)^{b}
W	5.60 (0.29)^{b}	1.50 (0.31)^{a}	0.00 (0.00)^{a}	0.00 (0.00)^{a}	0.00 (0.00)^{a}
EBW	5.60 (0.35)^{b}	3.34 (0.34)^{b}	3.75 (0.46)^{c}	2.00 (0.25)^{b}	1.00 (0.00)^{b}
EAW	4.60 (0.14)^{a}	2.00 (0.00)^{b}	2.00 (0.00)^{b}	0.00 (0.00)^{a}	0.00 (0.00)^{a}

Values = mean (SD). C= control; W= waterlogged; EBW=ethylene before waterlogged; EAW=ethylene after waterlogged. WAF= Weeks after flooding. Plants were three weeks old when treatment was applied and flooding introduced and sustained for four weeks. The values with similar alphabet as superscript on the same column do not differ significantly.

Supplementary Table 6. Stem girth (cm) of *Abelmoschus esculentus* plants subjected to waterlogged condition

Treatment	2WAF	4WAF	6WAF	8WAF	10WAF
Control	0.86 (0.16)[a]	1.02 (0.13)[a]	1.26 (0.15)[a]	1.43 (0.18)[a]	1.83 (0.12)[a]
W	0.74 (0.12)[a]	0.98 (0.19)[a]	1.26 (0.19)[a]	1.50 (0.17)[a]	1.73 (0.18)[a]
EBW	0.85 (0.11)[a]	0.99 (0.12)[a]	1.32 (0.14)[a]	1. 50 (0.11)[a]	1.85 (0.10)[a]
EAW	0.84 (0.11)[a]	0.91 (0.17)[a]	1.16 (0.19)[a]	1.36 (0.14)[a]	1.55 (0.10)[a]

Values = mean (SD). C= control; W= waterlogged; EBW=ethylene before waterlogged; EAW=ethylene after waterlogged. WAF= Weeks after flooding. Plants were three weeks old when treatment was applied and flooding introduced and sustained for four weeks. The values with similar alphabet as superscript on the same column do not differ significantly.

Supplementary Table 7. Stem girth (cm) of *Zea mays* plants subjected to waterlogged condition

Treatment	1WAF	3WAF	5WAF	7WAF	9WAF
Control	0.81 (0.14)[a]	0.96 (0.02)[a]	1.04 (0.15)[a]	1.04 (0.15)[b]	1.01 (0.00)[b]
W	0.84 (0.12)[a]	0.98 (0.19)[a]	0.00 (0.00)[a]	0.00 (0.00)[a]	0.00 (0.00)[a]
EBW	0.85 (0.11)[a]	0.99 (0.11)[a]	1.23 (0.05)[a]	1.27 (0.10)[b]	1.30 (0.10)[b]
EAW	0.93 (0.16)[a]	0.97 (0.11)[a]	1.20 (0.14)[a]	1.30 (0.12)[b]	0.00 (0.00)[a]

Values = mean (SD). C= control; W= waterlogged; EBW=ethylene before waterlogged; EAW=ethylene after waterlogged. WAF= Weeks after flooding. Plants were three weeks old when treatment was applied and flooding introduced and sustained for four weeks. The values with similar alphabet as superscript on the same column do not differ significantly.

Supplementary Table 8. Number of adventitious roots formed by *Abelmoschus esculentus* plants subjected to waterlogged condition

Treatment	5WAF	6WAF	7WAF
Control	0.00 (0.00)^{a}	0.00 (0.00)^{a}	3.00 (0.31)^{a}
W	2.00 (0.13)^{b}	2.00 (0.27)^{b}	4.00 (0.22)^{b}
EBW	2.00 (0.32)^{b}	4.00 (0.29)^{c}	5.00 (0.08)^{c}
EAW	2.00 (0.17)^{b}	4.00 (0.32)^{c}	5.00 (0.07)^{c}

Values = mean (SD). C = control; W= waterlogged; EBW=ethylene before waterlogged; EAW=ethylene after waterlogged. WAF= Weeks after flooding. Plants were three weeks old when treatment was applied and flooding introduced and sustained for four weeks. The values with similar alphabet as superscript on the same column do not differ significantly.

Supplementary Table 9. Number of adventitious roots formed by *Zea mays* plants subjected to waterlogged condition

Treatment	3WAF	4WAF	5 WAF
Control	0.00 (0.00)[a]	2.00 (0.00)[a]	2.00 (0.00)[b]
W	0.00 (0.00)[a]	2.00 (0.00)[a]	0.00 (0.00)[a]
EBW	2.00 (0.82)[b]	4.00 (2.12)[c]	6.00 (0.73)[d]
EAW	3.00 (0.31)[b]	3.00 (0.00)[b]	5.00 (0.71)[c]

Values = mean (SD). C= control; W= waterlogged; EBW=ethylene before waterlogged; EAW=ethylene after waterlogged. WAF= Weeks after flooding. Plants were three weeks old when treatment was applied and flooding introduced and sustained for four weeks. The values with similar alphabet as superscript on the same column do not differ significantly.

Supplementary Table 10. Distance (cm) of root-stem junction of *Abelmoschus esculentus* plants and the soil level under waterlogged condition

Treatment	5WAF	6WAF	7WAF
Control	0.00 (0.00)^{a}	0.00 (0.00)^{a}	0.30 (0.00)^{a}
W	0.10 (0.00)^{a}	0.25 (0.11)^{a}	0.50 (0.20)^{b}
EBW	0.20 (0.00)^{a}	1.45 (0.21)^{b}	1.62 (0.14)^{c}
EAW	0.17 (0.11)^{a}	0.32 (0.18)^{a}	0.56 (0.16)^{b}

Values = mean (SD). C = control; W= waterlogged; EBW=ethylene before waterlogged; EAW=ethylene after waterlogged. WAF= Weeks after flooding. Plants were three weeks old when treatment was applied and flooding introduced and sustained for four weeks. The values with similar alphabet as superscript on the same column do not differ significantly.

Supplementary Table 11. Distance (cm) of root-stem junction of *Zea mays* plants and the soil level under waterlogged condition

Treatment	3WAF	4WAF	5 WAF
Control	1.13 (0.25)^{a}	2.60 (0.30)^{c}	2.20 (0.36)^{c}
W	0.15 (0.07)^{a}	0.40 (0.00)^{a}	0.00 (0.00)^{a}
EBW	1.38 (0.23)^{b}	1.48 (0.31)^{b}	3.40 (0.17)^{c}
EAW	0.85 (0.21)^{a}	1.20 (0.14)^{b}	1.50 (0.00)^{b}

Values = mean (SD). C= control; W= waterlogged; EBW=ethylene before waterlogged; EAW=ethylene after waterlogged. WAF= Weeks after flooding. Plants were three weeks old when treatment was applied and flooding introduced and sustained for four weeks. The values with similar alphabet as superscript on the same column do not differ significantly. The 0.00 value was recorded for dead plants.

Supplementary Table 12. Average of number of flower buds formed by *Abelmoschus esculentus* plants under waterlogged condition

Treatment	10WAF	12WAF	14WAF
Control	0.00 (0.00) [a]	3.00 (0.40) [b]	4.00 (0.00) [b]
W	0.00 (0.00) [a]	2.00 (0.31) [a]	3.00 (0.00) [a]
EBW	0.00 (0.00) [a]	3.00 (0.00) [b]	4.00 (0.00) [b]
EAW	0.00 (0.00) [a]	2.00 (0.00) [a]	3.00 (0.41) [a]

Values = mean (SD). C = control; W= waterlogged; EBW=ethylene before waterlogged; EAW=ethylene after waterlogged. WAF= Weeks after flooding. Plants were three weeks old when treatment was applied and flooding introduced and sustained for four weeks. The values with similar alphabet as superscript on the same column do not differ significantly.

Supplementary Table 13. Average number of fruits formed by *Abelmoschus esculentus* plants under waterlogged condition

Treatment	13 WAF	14WAF	15 WAF
Control	3.00 (0.00) ^{c}	3.00 (0.00) ^{b}	4.00 (0.27) ^{b}
W	1.00 (0.00) ^{a}	2.00 (0.20) ^{a}	3.00 (0.31)^{a}
EBW	2.00 (0.00) ^{b}	3.00 (0.00) ^{b}	4.00 (0.00) ^{b}
EAW	2.00 (0.00) ^{b}	2.00 (0.00) ^{a}	3.00 (0.00)^{a}

Values = mean (SD). C = control; W= waterlogged; EBW=ethylene before waterlogged; EAW=ethylene after waterlogged. WAF= Weeks after flooding. Plants were three weeks old when treatment was applied and flooding introduced and sustained for four weeks. The values with similar alphabet as superscript on the same column do not differ significantly.

Supplementary Table 14. Soil bacterial assessment of different soil samples after plant growth under waterlogged condition

Soils	Heterotrophic bacterial counts after 48 hours ($\times 10^4$ cfu/g)	Bacterial isolates detected in the respective soil samples
C (Maize)	7.1	*Micrococcus varians, Micrococcus leutus,* and *Arthrobacter* sp.
W (Maize)	6.4	*Micrococcus leutus, Klebsiella* sp, *Serratia marcescens*
EBW (Maize)	9.7	*Arthrobacter* sp, *Serratia marcescens, Pseudomonas aeruginosa,*
EAW (Maize)	7.5	*Micrococcus varians, Micrococcus leutus, Pseudomonas aeruginosa*
C (Okra)	8.0	*Micrococcus leutus, Serratia marcescens, Arthrobacter* sp. *Pseudomonas aeruginosa* and *Klebsiella* sp.
W (Okra)	8.8	*Micrococcus leutus, Bacillus* spp and *Klebsiella* sp.
EBW (Okra)	7.6	*Serratia marcescens, Arthrobacter* sp, *Micrococcus leutus,*
EAW (Okra)	9.1	*Micrococcus varians, Micrococcus leutus, Bacillus* spp.

C = control; W = under waterlogged; EBW= ethylene priming before waterlogged; EAW= ethylene priming after waterlogged.
